# Special Issue: Non-Conventional Yeasts: Genomics and Biotechnology

**DOI:** 10.3390/microorganisms8010021

**Published:** 2019-12-20

**Authors:** Jürgen Wendland

**Affiliations:** Department of Microbiology and Biochemistry; Hochschule GEISENHEIM University, Von-Lade-Str. 1, 65366 Geisenheim, Germany; Juergen.Wendland@hs-gm.de

Non-conventional yeasts, i.e., the vast biodiversity beyond already well-established model systems such as *Saccharomyces cerevisiae, Candida albicans* and *Schizosaccharomyces pombe* and a few others, are a huge and untapped resource of organisms. These microorganisms have entered the scientific mainstage but still require the development of technologies and technical resources to speed up their further development.

This Special Issue on non-conventional yeasts adds to the effort to popularize novel yeasts and contains 10 papers covering a range of organisms and fields of application.

The use of *Kluyveromyces* in the conversion of a waste stream to bioethanol is described by Leandro et al. [1]. Dairy industry effluents contain a lot of energy that can be retained and converted to bioethanol by these yeast with great efficiency. Bioconversion of other organic wastes, mainly by *Yarrowia lipolytica*, is reviewed by Do et al. [2].

Beverage production using non-conventional yeasts is on the rise and also a key topic in this Special Issue. Previously regarded as spoilage yeasts, *Brettanomyces* nowadays gains interest for beverage innovations—particularly in the reduction of alcohol content—as described by Tiukova et al. [3]. This paper highlights the advances in genome sequencing and genome annotation that provide impulses in non-conventional yeast research. Another technological advance in the improvement of gene-targeting in lager yeasts is described by Bernardi et al. [4]. The role of mixed inocula in water kefir, an interesting fermented beverage, to control *Aspergillus flavus* growth is described by Gonda et al. [5]. Other non-conventional yeast, *Pichia kluyveri, Torulaspora delbrueckii* or *Lachancea thermotolerans*, were studied for their flavor contributions in sequential wine fermentations by Oliveira and Ferreira [6]. Here it was shown that these microorganisms could introduce higher amounts of acetate esters and medium chain fatty acid esters, all very interesting aromatic flavors in wine. A set of other yeasts, *Lachancea thermotolerans, Wickerhamomyces anomalus*, and *Zygotorulaspora florentina*, was used in beer co-fermentations by Canonico et al. [7]. An interesting observation was the general reduction in acetaldehyde content in these mixed fermentations.

Production of specific compounds by microorganisms is a strongly growing field in biotechnology. Xylitol production by *Meyerozyma, Spathaspora* and *Wickerhamomyces* was studied by Carneiro et al. [8]. The production of astaxanthin, a pigment and antioxidant, by a successfully engineered *Yarrowia lipolytica* strain was developed by Tramontin et al. [9]. The taxonomic distribution of cytochrome P450 monooxygenases, ubiquitous enzymes required for diverse metabolic reactions is reported by Linder [10]. This genomic survey describes the identification of eight P450 families that had not previously been reported in budding yeasts.

In summary, these papers describe a suite of applications of nonconventional yeasts in diverse biotechnological applications. They will further advance the field and stimulate growth in these areas.
